# Secretome Characterization and Correlation Analysis Reveal Putative Pathogenicity Mechanisms and Identify Candidate Avirulence Genes in the Wheat Stripe Rust Fungus *Puccinia striiformis* f. sp. *tritici*

**DOI:** 10.3389/fmicb.2017.02394

**Published:** 2017-12-11

**Authors:** Chongjing Xia, Meinan Wang, Omar E. Cornejo, Derick A. Jiwan, Deven R. See, Xianming Chen

**Affiliations:** ^1^Department of Plant Pathology, Washington State University, Pullman, WA, United States; ^2^School of Biological Sciences, Washington State University, Pullman, WA, United States; ^3^Wheat Health, Genetics, and Quality Research Unit, Agricultural Research Service, U.S. Department of Agriculture, Pullman, WA, United States

**Keywords:** avirulence genes, effectors, *Puccinia striiformis* f. sp. *tritici*, secretome, stripe rust, yellow rust

## Abstract

Stripe (yellow) rust, caused by *Puccinia striiformis* f. sp. *tritici* (*Pst*), is one of the most destructive diseases of wheat worldwide. Planting resistant cultivars is an effective way to control this disease, but race-specific resistance can be overcome quickly due to the rapid evolving *Pst* population. Studying the pathogenicity mechanisms is critical for understanding how *Pst* virulence changes and how to develop wheat cultivars with durable resistance to stripe rust. We re-sequenced 7 *Pst* isolates and included additional 7 previously sequenced isolates to represent balanced virulence/avirulence profiles for several avirulence loci in seretome analyses. We observed an uneven distribution of heterozygosity among the isolates. Secretome comparison of *Pst* with other rust fungi identified a large portion of species-specific secreted proteins, suggesting that they may have specific roles when interacting with the wheat host. Thirty-two effectors of *Pst* were identified from its secretome. We identified candidates for *Avr* genes corresponding to six *Yr* genes by correlating polymorphisms for effector genes to the virulence/avirulence profiles of the 14 *Pst* isolates. The putative *AvYr76* was present in the avirulent isolates, but absent in the virulent isolates, suggesting that deleting the coding region of the candidate avirulence gene has produced races virulent to resistance gene *Yr76*. We conclude that incorporating avirulence/virulence phenotypes into correlation analysis with variations in genomic structure and secretome, particularly presence/absence polymorphisms of effectors, is an efficient way to identify candidate *Avr* genes in *Pst*. The candidate effector genes provide a rich resource for further studies to determine the evolutionary history of *Pst* populations and the co-evolutionary arms race between *Pst* and wheat. The *Avr* candidates identified in this study will lead to cloning avirulence genes in *Pst*, which will enable us to understand molecular mechanisms underlying *Pst*-wheat interactions, to determine the effectiveness of resistance genes and further to develop durable resistance to stripe rust.

## Introduction

Wheat stripe (yellow) rust, caused by the obligate biotrophic fungus *Puccinia striiformis* Westend. f. sp. *tritici* Erikss. (*Pst*), is a significant threat to wheat production in the world (Chen, 2005; Wellings, 2011). Planting resistant cultivars is widely accepted as the most effective, economic, easy-to-use, and environmental-friendly way to control this disease (Chen, 2013). However, breeding resistant cultivars can be complicated by several biological characteristics of the pathogen, e.g., long-distance aerial dispersal of urediniospores and ever-changing virulent races (Brown and Hovmøller, 2002; Line, 2002; Chen, 2005; Liu et al., 2017). The rapid, long-distance aerial dispersal or unintentional introduction by travelers can lead to new invasions and reestablishments of the disease in previously disease-free or seasonally pathogen-absent regions (Wellings, 2011; Sharma-Poudyal et al., 2014). The rapid evolution of *Pst* results in “breakdown” of race-specific resistance after resistant cultivars are grown for only few years (Line and Qayoum, 1992; Bayles et al., 2000; de Vallavieille-Pope et al., 2012). In the United States, more than 200 different races have been identified since the 1960s with an average of 6 new races identified every year (Wan and Chen, 2014; Wan et al., 2016; Liu et al., 2017). The change in races has accelerated as more races, including new races have been detected since the year 2000. The new races, under favorable weather conditions, could shorten the life time of wheat cultivars with only race-specific resistance and cause destructive epidemics (Line and Qayoum, 1992; Chen, 2005; Chen et al., 2010; Hovmøller et al., 2016; Wan et al., 2016). Thus, understanding *Pst* evolution is critical and valuable not only for predicting and monitoring the population changes but also for developing cultivars with durable resistance for sustainable control of the disease. Therefore, the pathogenicity and molecular mechanisms that *Pst* uses to infect and interact with wheat need to be elucidated.

Although stripe rust is economically important, molecular mechanisms of *Pst*-wheat interactions are not fully understood. Based on the discoveries in other pathosystems, effectors, mainly protein molecules secreted from pathogens, are important components to modulate plant immunity and facilitate infection (Toruño et al., 2016). These effectors are located in different subcellular locations within plants, e.g., in the host cell and in the apoplastic space between adjacent cells, indicating their diverse functions ranging from modulating plant cellular metabolic pathways and signaling cascades, RNA silencing, anti-microbial inhibition and interfering with recognition machinery (Sharpee and Dean, 2016). To our knowledge, only a limited number of studies have been conducted to identify secreted protein (SP) genes and possible effectors in the *Pst* fungus (Yin et al., 2009, 2015; Huang et al., 2011; Saunders et al., 2012; Cantu et al., 2013; Cheng et al., 2015; Liu et al., 2016; Kiran et al., 2017). Among plant pathogen effectors, there is a special group, called avirulence (Avr) effectors which trigger multifaceted defense responses upon recognition by cognate plant resistance (R) proteins and therefore determine resistance specificity. The interactions between most *Avr* and corresponding *R* genes determine the gene-for-gene relationship in which “for each *R* gene that conditions resistance in the host there is a corresponding *Avr* gene that conditions pathogenicity in the parasite” (Flor, 1971). In fact, the first cloned effectors from rust and other fungi were all *Avr* effectors since it was easier to detect the avirulence function, e.g., the hypersensitive responses (Petre et al., 2014; de Wit, 2016). However, no Avr effectors have been identified and characterized in *Pst*.

The availability of *Pst* genomes and the advanced next generation sequencing technologies enable us to predict effector candidates from whole genome screening based on some conserved features of cloned fungi and fungi-like plant pathogen effectors, even though the cloned effectors generally show low sequence similarities among species (Sperschneider et al., 2015a). Such conserved features include secreted, small and cysteine-rich proteins which are representative for first cloned fungal Avr effector Avr9, effectors from *Cladosporium fulvum* and for most other cloned effectors (van Kan et al., 1991; Sperschneider et al., 2015c). Particularly in the flax rust fungus *Melampsora lini* (*Mli*), four *Avr* genes (*AvrL567, AvrM, AvrP4*, and *AvrP123*) were identified from 21 haustorially expressed secreted proteins (HESPs) and transient expression of these genes in flax caused resistance gene-mediated cell death, thus confirming their avirulence activities (Catanzariti et al., 2006). Haustoria are of special interest because it is a specialized infection structure through which the fungus obtains nutrients from the host and produces secreted effectors to interact with the host (Garnica et al., 2013, 2014). In the *Pst* fungus, Garnica et al. (2013) identified 437 HESPs as putative effectors by transcriptome sequencing. This result indicates that the mentioned conserved features are not one-size-fits-all criteria, and other rules could be added to the effector candidates mining pipeline. For example, the conserved structures of functionally characterized motifs also provide new opportunities for clustering and identifying plant pathogen effectors. More features, such as presence of long intergenic regions between effector genes, internal repeats and no PFAM domains except with known pathogenicity have been used in a comprehensive hierarchical clustering method to screen rust effectors (Saunders et al., 2012). Moreover, besides the criteria mentioned above, Cantu et al. (2013) incorporated the polymorphism analysis in haustoria-expressed genes into secretome characterization and identified five candidate effectors from 2,999 SPs for two closely related *Pst* isolates. However, no effectors were identified for particular avirulence genes.

In the present study, we characterized the *Pst* secretome and identified candidate effectors using meta-analysis of genome and transcriptome data using an advanced next generation sequencing technology and the latest pathogen-host interaction (PHI) database, e.g., PHI-base 4.0 to annotate *Pst* candidate effectors. We further identified candidate Avr effectors in *Pst* isolates corresponding to wheat resistance genes by correlating polymorphic variations with the virulence phenotypes of 14 *Pst* isolates. Previous studies of genetic inheritance of pathogenicity in flax-rust and stem rust-wheat pathosystems, and preliminary studies of *Pst*-wheat pathosystem suggested that the gene-for-gene model may also apply to *Pst*-wheat interactions (Flor, 1971; Tian et al., 2016; Yuan et al., 2017). Therefore, given that more than 70 permanently named and more than 60 provisionally named yellow rust (stripe rust) resistance (*Yr*) genes have been genetically characterized (Chen, 2013; McIntosh et al., 2013), we speculated that in the *Pst* genome there are hundreds of *Avr* genes encoding Avr effectors that are interacting specifically with corresponding *R* gene products following the gene-for-gene model. To screen for candidate effectors of *Avr* genes in *Pst*, we took advantages of the 18 *Yr* single-gene lines that are used to differentiate *Pst* races (Wan and Chen, 2014). By recording the incompatible or compatible reactions between each wheat line inoculated with the 14 selected *Pst* isolates, we were able to determine *Avr* (for avirulence) or *avr* (for virulence) in each *Pst* isolate corresponding to the 18 *Yr* genes. The virulence phenotype data were used to test the correlation with polymorphic effector genes for identifying candidates of the *Yr* genes. Our specific objectives were to (1) characterize the *Pst* secretome to identify candidate effectors by applying diverse screening criteria; (2) identify candidate *Avr* genes by correlation analysis of the virulence phenotypes and polymorphic effector genes; and (3) understand the virulence evolution of the *Pst* fungus.

## Materials and methods

### Selecting and virulence phenotyping *Pst* isolates

We selected 14 *Pst* isolates including seven previously sequenced isolates (Cantu et al., 2011, 2013; Cuomo et al., 2017). The other seven isolates were selected based on previous virulence data (Wan and Chen, 2014; Wan et al., 2016) to have relative balance of avirulent and virulent isolates for a maximum number of virulence loci. To confirm the virulence profiles, the 14 isolates were tested on the 18 wheat *Yr* single-gene differential lines, following the procedure described by Wan and Chen (2014). On each of the inoculated *Yr* single-gene line, the infection type (IT) of the isolate was recorded based on a 0–9 scale. Isolates with ITs 0–6 were considered as avirulent to the *Yr* single-gene line, while with isolates ITs 7–9 as virulent. To reduce phenotypic variation within avirulent and virulent classes, isolates with ITs 0–2 for avirulent reactions and with ITs 8–9 for virulent reactions were selected and thus intermediate ITs (3–7) were mostly avoided. Generally, the selected 14 Pst isolates represented a balanced virulent-avirulent profile to the majority of the *Yr* genes (Table 1).

**Table 1:** *Puccinia striiformis* f. sp. *tritici* isolates used in this study and their virulence/avirulence formulae.

**Isolate**	**Race**	**Origin**	**Year**	**Virulence (bold) and avirulence formula on *Yr* genes^a^**
09-134 (PST-127)	PSTv-11	US	2009	**1**,5,**6**,**7**,**8**,**9**,10,15,**17**,24,**27**,32,**43**,**44**,SP,Tr1,**Exp2**,**76**
11-281	PSTv-18	US	2011	1,5,6,7,8,9,10,15,17,24,27,32,43,44,SP,Tr1,Exp2,76
12-248	PSTv-2	US	2012	**1**,5,**6**,7,8,**9**,10,15,**17**,24,27,32,43,44,SP,Tr1,Exp2,**76**
12-346	PSTv-40	US	2012	1,5,**6**,**7**,**8**,**9**,**10**,15,17,**24**,**27**,**32**,**43**,**44**,SP,**Tr1**,**Exp2**,76
12-368	PSTv-4	US	2012	**1**,5,**6**,7,8,**9**,10,15,**17**,24,**27**,32,43,44,**SP**,Tr1,Exp2,**76**
PK08-2	PSTv-119	Pakistan	2008	1,5,**6**,**7**,**8**,9,10,15,**17**,24,**27**,32,**43**,**44**,SP,Tr1,**Exp2**,76
841541:430	360E137A	Australia	1984	1,**5**,6,7,8,9,10,15,17,24,27,32,43,44,SP,Tr1,Exp2,76
3-5-79 (PST-1)	PSTv-1	US	1979	**1**,5,6,7,8,9,10,15,17,24,27,32,43,44,SP,Tr1,Exp2,76
07-214 (PST-21)	PSTv-1	US	2007	**1**,5,6,7,8,9,10,15,17,24,27,32,43,44,SP,Tr1,Exp2,76
03-338 (PST-43)	PSTv-27	US	2003	1,5,6,7,8,9,**10**,15,**17**,**24**,27,**32**,43,44,SP,**Tr1**,Exp2,76
2K-041 (PST-78)	PSTv-35	US	2000	1,5,**6**,**7**,**8**,**9**,10,15,**17**,24,27,32,**43**,**44**,SP,**Tr1**,**Exp2**,76
08-220 (PST-127)	PSTv-11	US	2008	**1**,5**,6**,**7**,**8**,**9**,10,15,**17**,24,**27**,32,**43**,**44**,SP,Tr1,**Exp2**,**76**
07-168 (PST-130)	PSTv-69	US	2007	1,5,**6**,**7**,**8**,**9**,**10**,15,**17**,24,**27**,**32**,**43**,**44**,SP,Tr1,**Exp2**,76
09-001 (CYR-32)	PSTv-37	China	2009	1,5,**6**,**7**,**8**,9,10,15,**17**,24,**27**,32,**43**,**44**,SP,**Tr1**,**Exp2**,76

a *The Yr genes in bold are genes to which the isolate is virulent*.

### Whole genome sequencing, alignment, and genomic variation calling

Among the 14 isolates used in this study, 7 were sequenced previously using FLX chemistry with a Roche 454 and Illumina-adapted Fosmids by 454 Illumina (Cuomo et al., 2017); and Illumina (Cantu et al., 2011, 2013) sequencing approaches. Raw reads were downloaded from the National Center for Biotechnology Information (NCBI) Sequence Read Archive (SRA) or the Broad Institute Puccinia website (http://www.broadinstitute.org/) (Table 2). The remaining 7 isolates were sequenced in the present study using an Ion Proton platform. Genomic DNA was extracted from urediniospores using OmniPrep™ kit (G-Biosciences, St. Louis, MO). DNA libraries with 200-base-read for the Ion Proton™ system were prepared following the instruction of Ion Xpress™ Plus gDNA Fragment Library Preparation (Thermo Fisher Scientific Inc. 2014). The fragment size for each DNA library was checked using the Agilent®2100 Bioanalyzer® (Agilent Technologies, UK) instrument. Then the adaptors and barcodes were ligated to the sheared DNA. Fragment size selection of the library was conducted using E-Gel® SizeSelect™ agarose gel electrophoresis. The library was amplified following the Ion Xpress™ Plus gDNA Fragment Library Preparation protocol. Whole-genome sequencing was carried out using an Ion Proton™ system at the USDA-ARS Western Regional Small Grain Genotyping Laboratory at Pullman, WA. The chip for the Ion Proton™ system for this study was Ion PI™ Chip v2 which is able to ideally generate at least 10 Gb bases in one chip. On average, the isolates from Illuminia and Ion Proteon sequencing were sequenced at 26X and 32X coverage, respectively. The Ion Proton sequences of seven *Pst* isolates (09-134, 11-281, 12-248, 12-346, 12-368, PK08-2, and 841541:430) have been deposited in NCBI SRA under accession numbers SRR5050793, SRR5050794, SRR5050796, SRR5050790, SRR5050791, SRR5050792, and SRR5050795, respectively.

**Table 2:** Summary of mapping for variation calling from the genomic sequences of *Puccinia striiformis* f. sp. *tritici* isolates.

**Isolate^a^**	**Sequencing platform**	**NCBI SRA accession**	**Raw reads**	**Mapped reads**	**SNP**		**Gene**	
					**Homozygous**	**Heterozygous**	**Presence**	**Absence**
09-134	Ion Proton	SRR5050793	21,241,854	14,096,396	811,563	155,129	30	48
11-281	Ion Proton	SRR5050794	18,419,640	12,342,718	537,646	429,046	40	38
12-248	Ion Proton	SRR5050796	33,487,791	18,323,156	805,980	160,712	30	48
12-346	Ion Proton	SRR5050790	27,858,839	17,469,658	537,756	428,936	50	28
12-368	Ion Proton	SRR5050791	7,878,535	5,331,924	846,720	119,972	17	61
PK08-2	Ion Proton	SRR5050792	37,124,931	23,476,479	574,968	391,724	48	30
841541:430	Ion Proton	SRR5050795	7,952,069	5,539,275	633,325	333,367	45	33
3-5-79	Illumina	SRR172668, SRR172672	45,781,849	34,951,674	498,735	467,957	46	32
07-214	Illumina	SRR653741	36,328,044	26,678,202	507,879	458,813	39	39
03-338	Illumina	SRR769507	16,998,330	11,366,582	662,127	304,565	44	34
2K-041	Illumina	SRR987408	46,874,217	36,183,172	499,367	467,325	78	0
08-220	Illumina	SRR172671, SRR172673	45,138,107	32,900,503	741,482	225,210	50	28
07-168	Illumina	SRR058506	53,972,665	25,370,512	647,386	319,306	53	25
09-001	Illumina	SRR172667	39,727,649	28,825,195	502,934	463,758	52	26

a *The sequences of isolates by Ion Proton were from this study; the sequences of isolates 07-214 and 03-338 were from Cantu et al. (2013), the sequences of isolate 07-168 were from Cantu et al. (2011); and the sequences of remaining isolates (3-5-79, 2K-041, 08-220, and 09-001) were from the Broad Institute Puccinia website (http://www.broadinstitute.org/)*.

The adapter and barcode trimmed reads in the FASTQ format were corrected using Coral 1.4 (Salmela and Schröder, 2011) with sequencing technology set as −454 and other parameters as default. Quality assessment of corrected reads was conducted using the FASTQC program (Andrews, 2010). The reads that were shorter than 40 bp were discarded and all reads were cut to 246 bp by removing the 3′ low quality bases using Trimmomatic 0.33 (Bolger et al., 2014). The Illumina raw reads from NCBI SRA were also trimmed in such way. The high-quality trimmed reads in FASTQ format were used for genomic variants calling. The previously sequenced and assembled PST-78 genome (Cuomo et al., 2017) was used as a reference and a previously proposed framework by DePristo et al. (2011) for variation discovery was followed. The reference genome was indexed first and the high-quality trimmed reads from each isolate were mapped to the reference genome using bwa-mem algorithm with default setting in Burrows-Wheeler Alignment (BWA) Tool version 0.7.12 (Li and Durbin, 2010). The SAM formatted alignment information was converted to the BAM format after the duplicates were removed using SAMtools version 1.2 (Li, 2011). The BAM files were cleaned, validated and sorted using the Picard tools (version 1.129) (http://picard.sourceforge.net/), and then were indexed using SAMtools. To reduce the number of potentially mismatching insertion and deletion (Indels) in the alignment, the local realignment was conducted following two steps using Genome Analysis Toolkit (GATK) version 3.3 (van der Auwera et al., 2013). Firstly, the interval targets for local realignment were defined using GATK's RealignerTargetCreator with the default parameters. Secondly, GATK's IndelRealigner was used to perform Indel realignment of reads around defined targets from the previous step. The option -nWayout, with other default parameters, was used in this step to generate a BAM file for each isolate for further analyses. The BAM files were submitted to QualiMap v2.1 to evaluate the mapping quality (Okonechnikov et al., 2016). These analysis-ready BAM files were utilized in the following analyses. The first utilization was to call variants using GATK's HaplotypeCaller. We used VCFtools 0.1.12b to manipulate the variants in VCF format (Danecek et al., 2011). For correlation analysis, SNPs and Indels from only SP genes were used and filtered with the following criteria: –mac 3, –max-alleles 2 and –max-missing 1. The second use of the analysis-ready BAM files was to identify supercontigs with absence/presence polymorphism in each isolate. This analysis was achieved from the QualiMap results, and supercontigs were considered as absent in an isolate when no reads were mapped. The same procedure was applied to identify absence/presence polymorphic SPs. The analysis-ready BAM files were also used to generate secretome sequences for each isolate. To do so, a consensus genome sequence was generated for each isolate using the SAMtools mpileup function with -Q 10 and other default parameters. Then SP genes were extracted using the Bedtools getfasta function. These secretomes were used in the subsequent analyses to identify identical SPs among different *Pst* isolates.

### Principle component analysis

Whole-genome SNPs and InDels from section Whole Genome Sequencing, Alignment, and Genomic Variation Calling were separated into three regions, SP genes, non-SP genes, and non-coding regions. Principle component analysis (PCA) was conducted for each region using the “prcomp” function, and the results were visualized using the “scatterplot3d” package in R v3.2.3 (Ligges and Maechler, 2003; R Core Team, 2014).

### Correlation analysis

The polymorphisms obtained from previous section were used to identify potential correlation relationships with the virulence phenotypes corresponding to individual *Yr* genes. To facilitate correlation analysis, the SNP and Indel data in the VCF file were compressed and recoded using the BLINK program (http://zzlab.net/blink/index.html; parameters: –compress –vcf, and –recode –numeric, respectively) and the numeric format data were generated. The gene absence/presence polymorphisms were also coded as numeric. For phenotype data preparation, the non-polymorphic as well as low frequent traits were excluded. For example, all isolates used in this study were avirulent to *Yr15*, and only one isolate was virulent to *Yr5* and *YrSP*. Thus, these genes were excluded for correlation analysis. Also excluded were redundant traits. For example, all of the isolates virulent on *Yr7* were also virulent on *Yr8, Yr43, Yr44*, and *YrExp2*. By excluding these *Yr* genes, eight virulence traits were used for correlation analysis. The correlation analysis was conducted using the *cor* function in R version 3.2.3. The formulae used were:

$$\begin{array}{l} \;R=Cor\left(Y,X\right)\\ \;T=\;\frac{R}{\sqrt{\frac{1-R^{2}}{n-2}}}\\ p=2[1-Pt\left(\left|T\right|,n-2\right)] \end{array}$$

where *Y* and *X* were the phenotype and genotype matrix in numeric, respectively, n was the number of isolates, and *p* was the significance value. In this study, the Bonferroni corrected *p* significance, calculated as α/*m* in which *m* was the number of polymorphisms, was used as the threshold.

### Comparison of the genomes and secretomes of the wheat rust fungi

We started with three predicted proteomes of *Pst, P. graminis* f. sp. *tritici* (*Pgt*), and *P. triticina* (*Pt*). These three predicted proteomes were obtained from the *Puccinia* Group Sequencing Project, Broad Institute of Harvard and MIT (http://www.broadinstitute.org/). Then we followed a well-studied classical routing pipeline to predict secretomes (Saunders et al., 2012; Cantu et al., 2013). For each predicted proteome, the SignalP version 4.1, TargetP version 1.1, and TMHMM version 2.0 algorithms were used. SignalP was used to screen the presence and location of signal peptide cleavage sites, setting the options for eukaryotic organisms with other default parameters. The input files were protein sequences in the FASTA format. TargetP was used to confirm the presence of secretory pathway signal peptides and to remove the protein sequences with chloroplast transit peptides and mitochondrial targeting peptides. Protein sequences with transmembrane helices predicted using TMHMM were excluded from final protein data sets. The filtered protein sequences for each rust fungus were defined as the final secretome for subsequent analyses. We were aware that some SPs might be exported to the extracellular space through a non-classical mechanism in fungi, and therefore, the predicted secretomes using this classical method in this study might not represent the entire secretome repertoires in the rust fungi.

The complete sets of proteomes were used to make comparisons among the three wheat rust fungi. Realizing the difficulties arising from the presence of paralogs, program Proteinortho version 5.11 was used to detect orthologs and co-orthologs (Lechner et al., 2011). The blastp function was performed using NCBI BLAST version 2.2.24+, which was installed as a part of the Proteinortho package. All analyses with Proteinortho were conducted with the default values except the following: *E*-value for blast was set as 1e-03, and the minimum coverage of best blast alignments in percent was set as 20. Also, selfblast and singles were applied to directly detect paralogs and to report unique sequences in each proteome, respectively. It should be noted that the *E*-value of 1e-03 was set arbitrarily, fewer species-specific proteins would be obtained if *E*-value was set as 1e-05 or even lower. The same analyses were conducted using the secretomes of the three wheat rust fungi.

The phylogenomic relationships were estimated from six selected rust fungi including *Pst, Pgt, Pt, Melampsora larici-populina* (*Mlp*) (the leaf rust fungus of popular, NCBI GenBank assembly accession GCA_000204055.1), *M. lini* (*Mli*) (the flax rust fungus) (Nemri et al., 2014) and *Septobasidium* spp. (*Sep*) (http://genome.jgi.doe.gov/Sepsp1/Sepsp1.home.html). Orthologs and paralogs among these six proteomes were detected using program Proteinortho version 5.11 (*E*-value as 1e-05, identity as 50%, and minimum coverage as 100%). The orthologous groups from the Proteinortho output were kept only when all six species were covered so that the protein absence/presence was not included for phylogenomic analyses. For each orthologous group only a single protein sequence was selected from each proteome and used for alignment. For each group, the six orthogous proteins were kept in one multi-FASTA file and used as input for multiple protein alignments using the CLUSTAL Omega algorithm (Sievers et al., 2011) with default parameters. Subsequently, the 63 alignment files in NEXUS format were concatenated using FASconCAT v1.0 (Kück and Meusemann, 2010). Phylogenomic inference was conducted using Bayesian-based method in program MrBayes v3.2.6 (Ronquist et al., 2012). A partitioned analysis was set up for MrBayes so that different models could be estimated and applied for each protein. Our data set was divided into 63 partitions as each protein alignment was considered as one partition. Evolutionary models were selected based on ProtTest 3.4 (Darriba et al., 2011). The best-fit model was incorporated into MrBayes as parameters and priors for each protein sequence. Then the parameters were unlinked so that each partition has its own set of parameters. We allowed all our partitions to evolve under different rates by setting overall rate to be different across partitions using commands prset applyto = (all) and ratepr = variable. Other parameters including model parameters, priors and the Markov Chain Monte Carlo sampling parameters were set as defaults. The performance was stopped when average standard deviation of split frequencies was lower than 0.01 and the performance was confirmed by checking the stationarity of the plot of the generation vs. the log probability of the data between two separated runs. Totally, four chains of 200,000 generations were run.

### *Pst* secretome characterization and annotation

**Figure 1:**
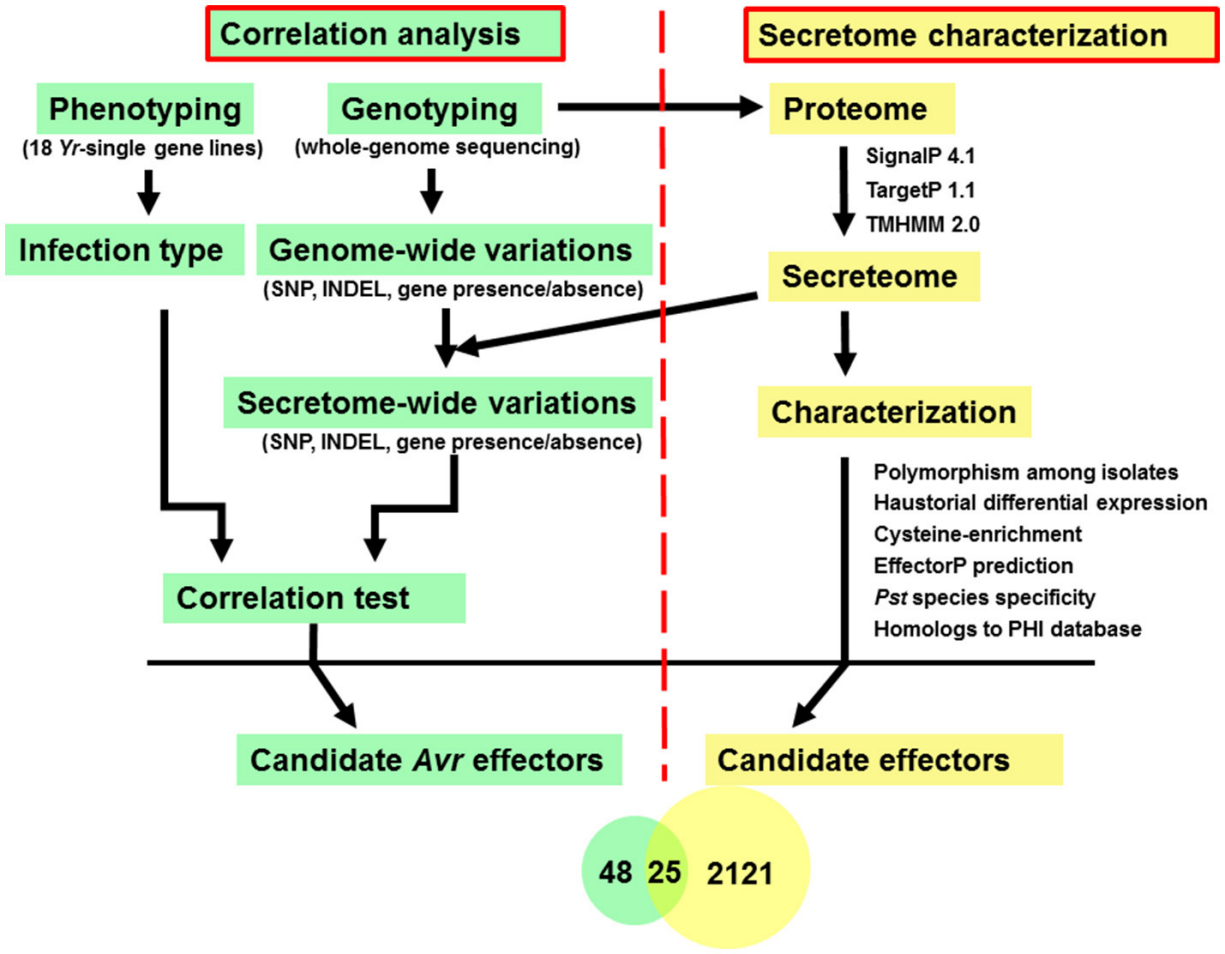
Analysis pipeline for identification of candidate *Avr* effectors in wheat stripe rust fungus *Puccinia striiformis* f. sp. *tritici*. Left: Correlation analysis between phenotypes, which were recorded as infection type by testing each of the *Pst* isolates on 18 *Yr* single-gene lines, and genotypes, which were conducted by whole-genome sequencing, to identify candidate *Avr* effectors corresponding to each avirulence gene. Right: Secretome characterization of a reference genome, PST-78. Secreted proteins were predicted using a classic routine pipeline from the well-annotated PST-78 proteome (http://www.broadinstitute.org/). Then each secreted protein was characterized using multiple criteria including whether it is (a) polymorphic among isolates; (b) haustorial differentially expressed; (c) cysteine-rich; (d) predicted to be effector using program EffectorP; (e) *Pst* species specific; and (f) homologous to known effectors by searching the PHI database.

In this study, diverse characters were applied to the *Pst* secretome which was predicted from the previous section, and a general analysis pipeline was shown in Figure 1. These characters included (a) typical fungal effector features, e.g., secreted, protein length, number and percent of cysteine residues; (b) haustorially specific expression; (c) effector prediction by program EffectorP; (d) polymorphisms among *Pst* isolates; and (e) the correlation relationships with *Yr* genes.

To determine how many and which SPs were differentially expressed in haustoria, two sets of previously published HESPs were retrieved from Cantu et al. (2013) and Garnica et al. (2013). DE Seq *p*-value of 0.05 was used as a cut-off to set HSPs from Cantu et al. (2013). These HESPs were blasted against the PST-78 secretome using BLASTP with *E*-value set as 1e-10 and similarity as 90. Then we tested whether the SPs were identical or polymorphic among the sequenced isolates. To reduce the computational load, only five *Pst* isolates were used to identify identical protein sequences, and the incomplete proteins were excluded from the proteomes. These five *Pst* isolates, PK08-2, 2K041, 3-5-79, 12-248, and 09-001, were selected based on their mapping depth to avoid false polymorphisms caused by sequencing. The proteome was translated from the consensus genome sequence obtained from previous section for each isolate with the PST-78 gff3 annotation file using the gffread function in Cufflinks v2.2.1 (Trapnell et al., 2010). Each of these 6 proteomes was blasted against the remaining 5 proteomes using NCBI BLAST version 2.2.24+ with *E*-value set as 1e-10. The BLSAT results were filtered manually. The proteins were considered identical only when (1) the whole length of the protein was covered in the BLAST alignment, (2) no mismatches and (3) no gaps in the alignment. As expected, some of the proteins were only identical among several of these 6 isolates. In this case, only proteins that were identical among all 6 isolates were considered as highly conserved proteins in *Pst*. To predict effectors, the PST-78 predicted secretome was submitted to program EffectorP with default parameters (Sperschneider et al., 2015b).

The PST-78 predicted secretome was annotated using Blast2GO v3.3 (Conesa et al., 2005). LocTree3 (Goldberg et al., 2014), and NucPred (Brameier et al., 2007) were used to predict subcellular localizations. NLStradamus (Nguyen et al., 2009) was used to predict Nuclear Localization Signals. PFAM domains were annotated using HMMSCAN searches against the PFAM database in HmmerWeb v2.3.3 (Finn et al., 2015). To determine the putative functions of SPs from *Pst*, similar to known fungal effectors, BlastP was used to search against PHI-base 4.0 (Urban et al., 2015). The secretome was scanned to search the effector motifs [L/I]XAR, [R/K]CXXCX12H, RXLR, [Y/F/W]XC, YXSL[R/K], and G[I/F/Y][A/L/S/T]R between amino acids 10–110 using FIMO version 4.11.2, with a *p*-value of 1e-04 (Grant et al., 2011).

## Results

### Defining the *Puccinia* secretome

**Figure 2:**
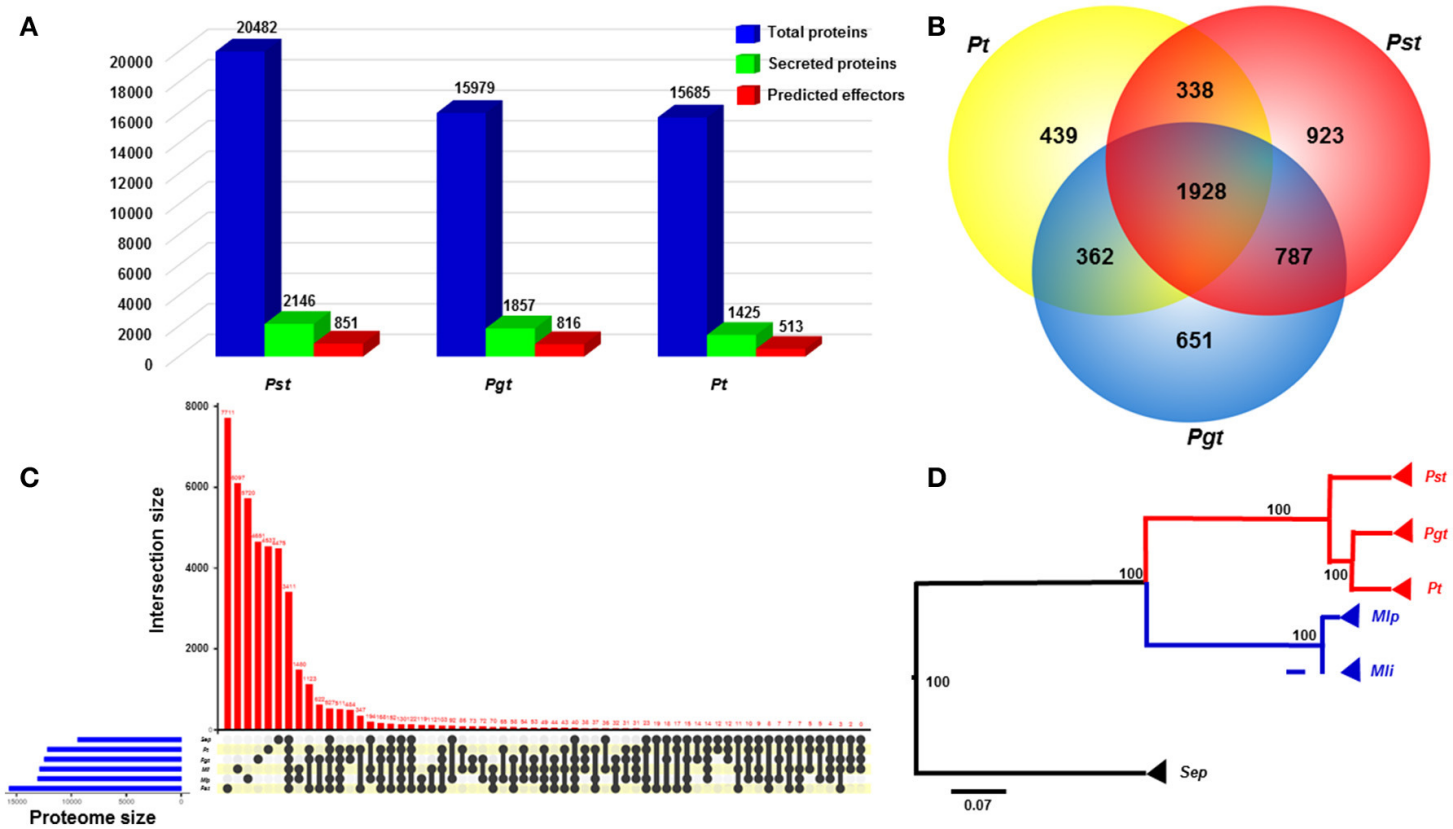
Comparative genomics analyses between *Puccinia striiformis* f. sp. *tritici* (*Pst*) and other rust fungi. **(A)** Comparison of the numbers of total proteins, secreted proteins and predicted effectors among three wheat rust fungi, *Pst, Pgt* (*P. graminis* f. sp. *tritici*), and *Pt* (*P. triticina*), indicated a large portion of secreted proteins in the three wheat rust fungi. The predicted effectors were defined as effectors predicted from a machine learning-based program EffectorP. **(B)** Venn diagram comparing the secretome with paralogs among the three rust fungi. A large proportion of species-specific secreted proteins was suggested in each of the three rust fungi. **(C)** Comparison of complete proteomes between *Pst* and five other Basidiomycete rust fungi. The comparison was visualized using package UpSetR in R v3.2.3. **(D)** A rooted phylogenetic tree showing evolutionary relationships of *Pst* with other five rust fungi. The phylogram suggested the early divergence of *Pst* from the most recent common ancestor of wheat rust fungi. The phylogenic relationship was estimated based on 63 orthologous protein sequences using MrBayes v3.2.6. Bootstrap values were shown. Scale bar indicted an evolutionary distance of 0.07 amino acid substation per position in the sequence.

We defined and compared the secretomes and effector repertoires of three wheat rust fungi following a classical pipeline in which SPs were predicted based on the presence of N-terminal signal peptides and the absence of transmembrane helices (Figure 1). The PST-78 secretome was predicted from the previously assembled and annotated proteome (20,482 proteins) available at the Puccinia genome database of the Broad Institute (http://www.broadinstitute.org/). The predicted PST-78 secretome contains 2,146 SPs, representing 10.48% of all proteins. The *Pst* secretome was similar to that of other wheat rust fungi *Pgt* and *Pt* in the percentage of SPs out of the total number of proteins. In contrast, the *Pgt* secretome comprised of 1,857 SPs (11.62%) over the total of 15,979 proteins, while the *Pt* secretome comprised of 1,425 SPs (9.08%) over the total of 15,685 proteins (Figure 2A). The proportions of SPs fall in the higher part of the range of the general proportion range (4–14%) of fungi (Lowe and Howlett, 2012).

### Comparative genomics showing *Pst*-specific SP effectors

We conducted a comparison analysis to estimate the specificity of SPs among three wheat rusts. In this analysis, only SPs from *Pst, Pgt*, and *Pt* were used, resulting in a total of 5,428 proteins analyzed. Among these proteins, 1,928 proteins (in 444 orthologous families) were conserved among the three rust fungi (Figure 2B). All three rust fungi have a large number of species-specific SPs (SSSPs), with *Pst* having the highest proportion of 43.01% (923; in 747 orthologous families), followed by *Pgt* having 35.05% (651; in 523 orthologous families) and *Pt* having 30.80% (439; in 384 orthologous families) (Figure 2B and Supplementary Table 1). The numbers of SSSP for the three Puccinia species are comparable as the number for *Pt* is very close to the number of 420 candidate secreted effector proteins (CSEPs) specific for *Pt* (Cuomo et al., 2017). When we applied a broader comparison including the proteins of additional Pucciniomycetes proteins mentioned in the methods, the proportion of SSSPs were reduced to 34.16% (733) in *Pst*, 27.08% (503) in *Pgt*, and 22.38% (319) in *Pt*. The decrease in the number of SSSPs might be explained by: (i) some of them had non-secreted orthologs in other rust fungi, or (ii) some are specific among wheat rust fungi, but have orthologs in other phylogenetically distant rust fungi. Nevertheless, the reduced numbers of SSSPs are still big, and the SPs might have specific roles in each species.

We also conducted comparative genomic analysis between *Pst* and a set of Pucciniomycetes fungi. Based on orthologous analysis, a large proportion of phytopathogenic rust fungi proteins were found to be species-specific (Figure 2C; Lex et al., 2014). For example, *Pst* has 9,108 (44.46%) unique proteins grouped in 7,711 orthologous families (Supplementary Table 2). In contrast, the numbers of species-specific proteins were lower in *Pt* (5,358 or 34.16% in 4,537 orthologous families) and *Pgt* (5,394 or 33.75% in 4651 orthologous families) (Figure 2C, Supplementary Tables 2). The higher number of unique proteins in *Pst* than those in *Pt* and *Pgt* may reflect the relatively distant phylogenetically relationship between *Pst* and the other two wheat rust fungi, as shown in Figure 2D, which was inferred from 63 conserved protein sequences among six Pucciniomycetes fungi. The earlier divergence of *Pst* from *Pgt* and *Pt* was consistent with previous estimation from phylogenetic analysis of 1,208 single-copy orthologs (Cuomo et al., 2017).

### Genome sequencing, variations and genetic diversity of *Pst* isolates

In addition to the 7 previously sequenced isolates, we selected 7 *Pst* isolates for Ion Proton sequencing with the aim to generate a balanced virulence-avirulence profile for correlation study. A summary of the whole genome sequencing data for the 7 isolates (09-134, 11-281, 12-248, 12-346, 12-368, PK08-2, and 841541:430) produced by Ion Proton is shown in Table 2. Also shown in Table 2 are the sequence summary data for the previously sequenced 7 isolates (3-5-79, 07-214, 03-338, 2K-041, 08-220, 07-108, and 09-001) by Illumina.

**Figure 3:**
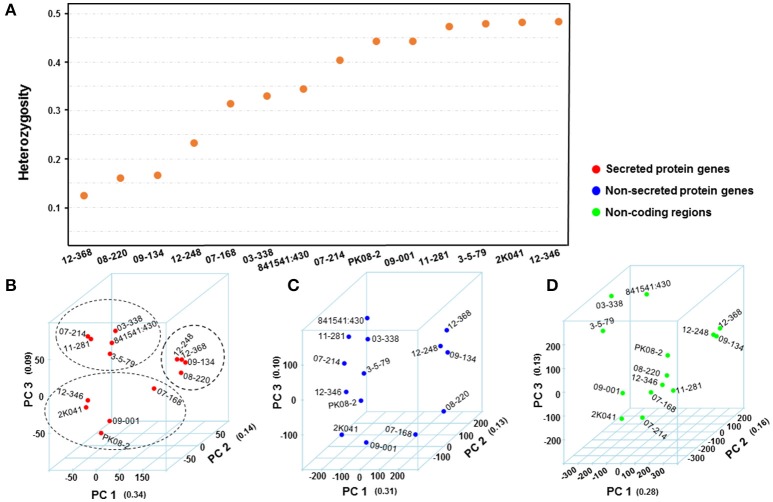
Comparison of *Puccinia striiformis* f. sp. *tritici* (*Pst*) genetic diversity from different genomic regions. **(A)** Distribution of mean heterozygosity over secreted protein (SP)-coding regions for each isolate. The heterozygosity was calculated first as He/(He+Ho), where He and Ho are the number heterozygous and homozygous SNP and Indel sites, respectively. **(B**–**D)** 3-dimentional plots of principle component analyses (PCA) using genomic variations from SP, non-SP, and non-coding gene regions of 14 *Pst* isolates. The number in quotes along each principle component (PC) is the proportion of variance explained by the PC. The values along each axis are eigenvalues calculated for isolates by PCA.

The reads of each isolate were aligned to the PST-78 reference genome to call SNPs and Indels. During filtering, SNPs and Indels were kept only when minor alleles appeared at least three times (–mac 3 in VCFtools, see Methods). Totally, 48,059 high quality variants including 41,764 SNPs and 6,295 Indels from SP genes were kept for subsequent analyses. To measure genetic diversity, we calculated the rate of He/(Ho+He), in which He and Ho represented heterozygous sites and homozygous sites, respectively. Generally, different isolates showed different heterozygosity rates (Figure 3A). We noticed that the isolates with low heterozygosity had a wider virulence spectrum compared with highly heterozygous isolates, which is consistent with the observations from our previous population study (Xia et al., 2016a).

We performed principal component analysis (PCA) to evaluate the differences between secreted and non-secreted protein gene variants and variants in non-coding regions in dissecting the correlational structure. As shown in Figures 3B–D, the SNPs and Indels in SP gene regions had a potential to reveal hidden population structures, while the variants in non-SP gene and non-coding regions did not. For instance, the four isolates (08-220, 09-134, 12-368, and 12-248) with low heterozygosity and broad virulence profiles were clustered together in Figure 3B, and the five less virulent and highly heterozygous isolates (03-338, 07-214, 11-281, 3-5-79, and 841541:430) were also clustered together, whereas such patterns were not seen in Figures 3C–D. Considering that the revealed structure in Figure 3B associated with the virulence profile in Table 1, we would hypothesize that SP genes may contribute more to the virulence variability.

### Prediction of *Pst* effectors

**Figure 4:**
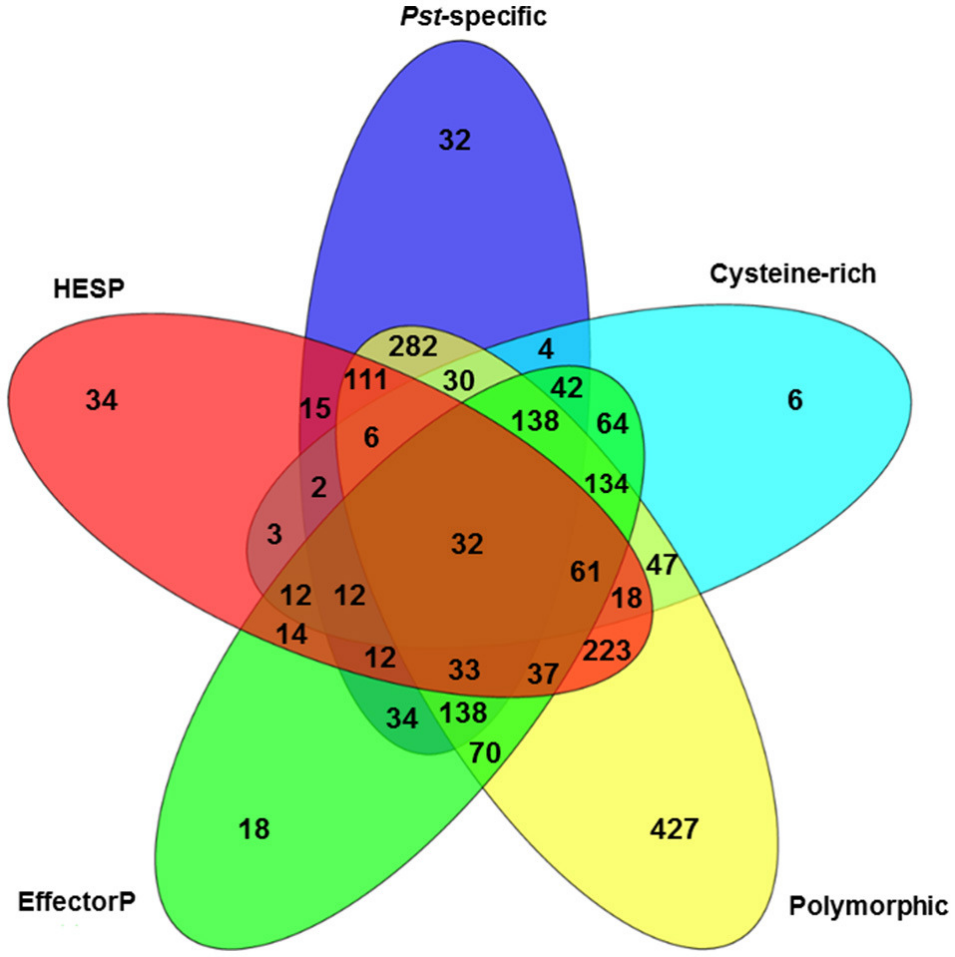
Characterization of the *Puccinia striiformis* f. sp. *tritici* (*Pst*) secretome using five criteria: cysteine-rich, polymorphic among *Pst* isolates, EffectorP, haustorially expressed secreted protein (HESP) and *Pst*-specific.

To further characterize the *Pst* secretome and predict effectors, the 2,146 predicted SPs were evaluated using five criteria. First, 6 *Pst* secretomes were compared to determine the SPs that were polymorphic among these different isolates. By doing this, we assumed that the virulence-related proteins should be polymorphic since these isolates had different virulence-avirulence profiles. In total, 1,787 (83.27%) out of the 2,146 SPs were polymorphic (Figure 4). Secondly, we identified 923 SPs as *Pst*-specific among the three wheat rust causing species. Details of this group are illustrated in the next section. Thirdly, we used a cutoff of at least 3% of cysteine in the SP sequences as a criterion and identified 611 SPs. Fourthly, we retrieved a total of 2,154 haustorial differentially expressed (HDE) proteins from previous studies and identified 625 of *Pst* SPs to be homologous to these HDE proteins. Lastly, we used a machine learning-based program, EffectorP, to predict *Pst* putative effector proteins from SPs. A total of 851 (39.7%) of 2,146 *Pst* SPs were predicted to be effectors. For comparison, we also conducted the same analysis for *Pgt* and *Pt*, and predicted 816 (43.9%) of 1,857 SPs as effectors in *Pgt* and 513 (36.0%) of 1,425 SPs as effectors in *Pt*. It should be noted that most of the SPs that passed one criteria also satisfied at least one of other criteria, the group comprising of polymorphic SPs was the only exception. For example, over 96% (891 out of 923) of *Pst-*specific SPs had at least one of the other criterion (Figure 4). Taking all of the five criteria together, 32 *Pst* SPs were identified to be highly possible candidate effectors.

### SP genes significantly correlated with avirulence genes in *Pst*

**Figure 5:**
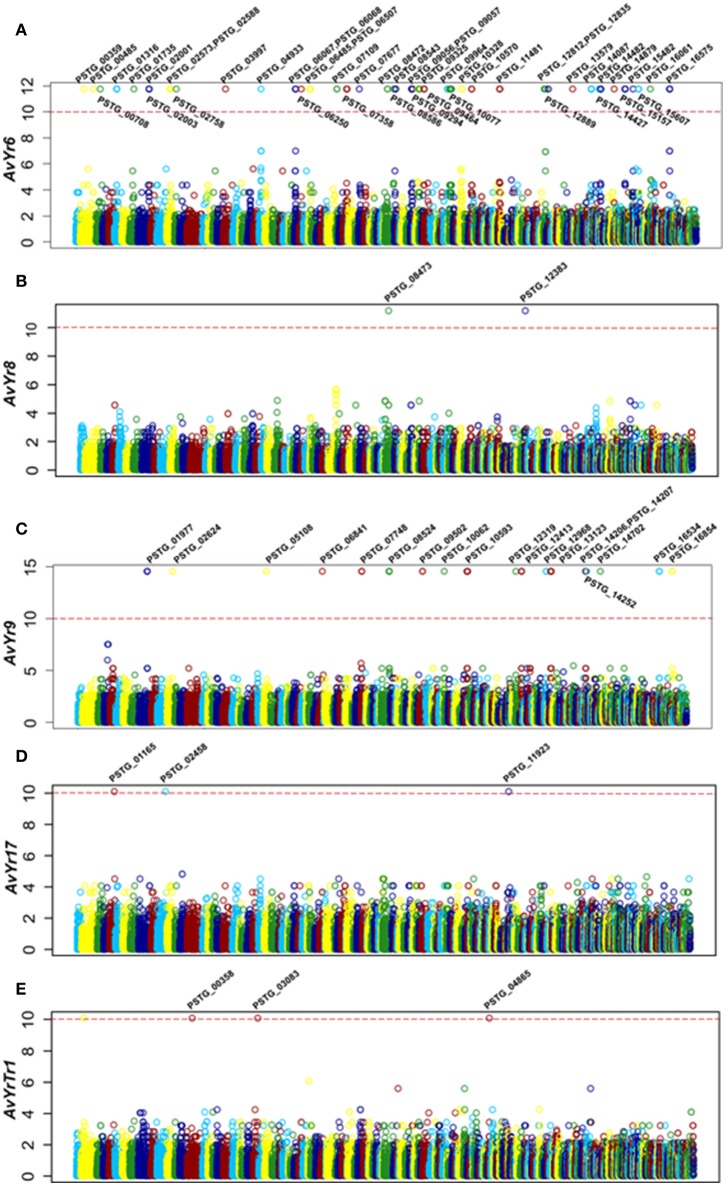
Correlation analysis identified secreted protein genes associated with *Avr* genes. **(A**–**E)** Manhattan plots showing the correlation analysis results, with each plot representing candidate *Avr* genes corresponding to a *Yr* gene. X-axis represents the genomic position of SNPs or Indels at the supercontigs of the PST-78 reference genome, and Y-axis is -log_10_ transformed significance *P* values. SNPs and Indels are represented as empty circles. The dashed red lines are Bonferroni-corrected threshold. The gene name in the significant correlation is also shown.

Since previous genome-based effectors mining studies have not emphasized avirulence effectors (Saunders et al., 2012; Cantu et al., 2013), we conducted a correlation analysis of effector polymorphisms and avirulence phenotypes. The avirulence function of an effector can be inferred, based on the gene-for-gene concept, from the incompatible interaction between the pathogen and host. In the *Pst*-wheat pathosystem, the presence of an *Avr* gene in the isolates is indicated when they are avirulent to the corresponding *Yr* gene. Based on this rationale, we conducted a correlation analysis to identify *Avr* candidates corresponding to 18 *Yr* genes. Toward this goal, we selected 14 *Pst* isolates, confirmed their virulence/avirulence profiles for each *Yr* gene, and conducted the correlation analysis. We identified 73 SP or effector genes that were significantly correlated with the avirulenc/virulence patterns to 5 *Yr* genes, *Yr6, Yr8, Yr9, Yr17*, and *YrTr1* (Figures 5A–E). Features of these effectors were shown in Table 3, and details were available in Supplementary Table 3.

**Table 3:** Candidate effectors for avirulence genes in *Puccinia striiformis* f. sp. *tritici*.

**Protein ID**	**Length (aa)**	**Cysteine**	**Haustorial differentially expressed**	**Subcellular location**	**Homology to (e-value)**	**PFAM domain**	**Note**
***AvYr6*** **CANDIDATES**							
PSTG_00485	130	4	No	Secreted	–	Lytic transglycolase (PF03330)	–
PSTG_02758	190	8	No	Secreted	–	–	Motif [Y/F/W]XC
PSTG_06067	368	2	Yes	Nucleus	–	–	–
PSTG_06250	204	4	No	Vacuole	–	ML domain (PF02221)	*Pst* specific
PSTG_07109	191	7	Yes	Secreted	–	–	–
PSTG_08586	86	7	No	Secreted	–	–	*Pst* specific
PSTG_09464	279	4	No	Mitochondrion	*Zymoseptoria tritici* ZtCBR1 (2.02e-82)	Oxidoreductase FAD-binding domain (PF00970,PF00175)	Reduced virulence in *Z. tritici* (Derbyshire et al., 2015)
PSTG_10570	120	6	No	Secreted	–	–	*Pst* specific
PSTG_12812	125	4	No	Secreted	–	–	Motif RSIVEQD
PSTG_12835	265	5	Yes	Secreted	–	Lipase (class 3) (PF01764)	–
PSTG_12889	259	10	No	Secreted	–	–	–
PSTG_13579	143	9	No	Secreted	–	–	*Pst* specific
PSTG_14087	293	10	Yes	Secreted	–	–	–
PSTG_15607	220	5	Yes	Secreted	–	–	–
PSTG_15157	102	6	No	Secreted	–	–	*Pst* specific
PSTG_15482	152	8	No	Secreted	–	–	–
PSTG_16061	232	7	Yes	Secreted	–	CFEM domain (PF05730)	Fungal pathogenesis (Kulkarni et al., 2003)
***AvYr8* CANDIDATES**							
PSTG_08473	215	6	No	Plasma membrane	*Xanthomonas campestris* pv *vesicatoria* CaRING1 (2.74e-6)	Ring finger domain (PF13639)	Required for cell death and the salicylic acid-dependent defense response (Lee et al., 2011)
PSTG_12383	446	5	Yes	Secreted	*Verticillium fungicola* vfglu1 (3.67e-19)	Cellulase (glycosyl hydrolase family 5) (PF00150)	Reduced virulence in *V. fungicola*
***AvYr9*** **CANDIDATES**							
PSTG_05108	546	7	Yes	Nucleus	–	–	*Pst* specific
PSTG_08524	185	1	Yes	Nucleus	–	–	Motif RXLR
PSTG_12319	111	7	Yes	Secreted	–	–	*Pst* specific
PSTG_14207	238	4	Yes	Mitochondrion	–	–	–
PSTG_16854	674	8	No	Nucleus	*Ustilago maydis* fuz7 (5.08e-156)	Protein kinase domain (PF00069)	Virulence factor in *U. maydis* (Lanver et al., 2010)
***AvYr76*** **CANDIDATE**							
PSTG_18147	102	8	No	Secreted	–	–	*Pst* specific; Gene presence/absence polymorphism
**CANDIDATES SHOWING HIGH SIMILARITY TO PHI DATABASE**							
PSTG_09480	298	7	No	Secreted	*Phytophthora_sojae* XEG1 (9.27e-14)	Glycosyl hydrolase family 12 (PF01670)	Plant avirulence determinant in *P. sojae* (Ma et al., 2015)
PSTG_09484	294	6	No	Secreted	*Phytophthora_sojae* XEG1 (4.75e-16)	Glycosyl hydrolase family 12 (PF01670)	Plant avirulence determinant in *P. sojae* (Ma et al., 2015)
PSTG_10154	266	11	Yes	Secreted	*Magnaporthe oryzae* ACE1 (7.26e-3)	–	Plant avirulence determinant in *M. oryzae* (Böhnert et al., 2004)
PSTG_02725	242	11	Yes	Endoplasmic reticulum	*Xanthomonas campestris* pv *vesicatoria* CaRING1 (4.96e-5)	Ring finger domain (PF13639)	Required for cell death and the salicylic acid-dependent defense response (Lee et al., 2011)
PSTG_12818	278	14	No	Endoplasmic reticulum	*Xanthomonas campestris* pv *vesicatoria* CaRING1 (4.88e-5)	Ring finger domain (PF13639)	Required for cell death and the salicylic acid-dependent defense response (Lee et al., 2011)
PSTG_14686	228	7	Yes	Plasma membrane	*Xanthomonas campestris* pv *vesicatoria* CaRING1 (6.25e-5)	Ring finger domain (PF13639)	Required for cell death and the salicylic acid-dependent defense response (Lee et al., 2011)
PSTG_15141	250	12	Yes	Plasma membrane	*Xanthomonas campestris* pv *vesicatoria* CaRING1 (2.32e-4)	Ring finger domain (PF13639)	Required for cell death and the salicylic acid-dependent defense response (Lee et al., 2011)
PSTG_01920	204	4	No	Cytoplasm	*Candida_albicans* SOD5 (1.76e-18)	Copper/zinc superoxide dismutase (PF00080)	Loss of pathogenicity in *C. albicans* (Martchenko et al., 2004)
PSTG_04011	343	5	No	Peroxisome	*Candida_albicans* SOD5 (1.02e-13)	Copper/zinc superoxide dismutase (PF00080)	Loss of pathogenicity in *C. albicans* (Martchenko et al., 2004)
PSTG_07834	196	8	No	Cytoplasm	*Candida_albicans* SOD5 (1.02e-13)	Copper/zinc superoxide dismutase (PF00080)	Loss of pathogenicity in *C. albicans* (Martchenko et al., 2004)
PSTG_10359	210	6	Yes	Peroxisome	*Candida_albicans* SOD5 (1.47e-7)	Copper/zinc superoxide dismutase (PF00080)	Loss of pathogenicity in *C. albicans* (Martchenko et al., 2004)
PSTG_10364	178	7	Yes	Cytoplasm	*Candida_albicans* SOD5 (1.92e-8)	Copper/zinc superoxide dismutase (PF00080)	Loss of pathogenicity in *C. albicans* (Martchenko et al., 2004)
PSTG_11365	176	7	Yes	Cytoplasm	*Candida_albicans* SOD5 (3.52e-7)	Copper/zinc superoxide dismutase (PF00080)	Loss of pathogenicity in *C. albicans* (Martchenko et al., 2004)
PSTG_11369	186	8	No	Cytoplasm	*Candida_albicans* SOD5 (2.55e-12)	Copper/zinc superoxide dismutase (PF00080)	Loss of pathogenicity in *C. albicans* (Martchenko et al., 2004; Gleason et al., 2014)
PSTG_15532	179	5	No	Cytoplasm	*Candida_albicans* SOD5 (8.44e-11)	Copper/zinc superoxide dismutase (PF00080)	Superoxide dismutase in *C. albicans* (Martchenko et al., 2004)
PSTG_04010	187	5	No	Cytoplasm	*Candida_albicans* SOD5 (1.24e-11)	Copper/zinc superoxide dismutase (PF00080)	Superoxide dismutase in *C. albicans* (Martchenko et al., 2004)
PSTG_09596	204	5	No	Secreted	*Candida_albicans* SOD5 (6.51e-6)	Copper/zinc superoxide dismutase (PF00080)	Superoxide dismutase in *C. albicans* (Martchenko et al., 2004)
PSTG_10366	193	4	No	Secreted	*Candida_albicans* SOD5 (1.54e-5)	Copper/zinc superoxide dismutase (PF00080)	Superoxide dismutase in *C. albicans* (Martchenko et al., 2004)
PSTG_11367	192	4	No	Mitochondrion	*Candida_albicans* SOD5 (1.08e-5)	Copper/zinc superoxide dismutase (PF00080)	Superoxide dismutase in *C. albicans* (Martchenko et al., 2004)
PSTG_11370	186	4	No	Cytoplasm	*Candida_albicans* SOD5 (1.04e-11)	Copper/zinc superoxide dismutase (PF00080)	Superoxide dismutase in *C. albicans* (Martchenko et al., 2004)
PSTG_02563	159	3	Yes	Cytoplasm	*Botrytis cinerea* BcFKBP12 (6.80e-19)	FKBP-type peptidyl-prolyl cis-trans isomerase (PF00254)	Sulfur regulation and pathogenic development in *B. cinerea* (Meléndez et al., 2009)
**CANDIDATES WITH KNOWN EFFECTOR MOTIFS**							
PSTG_00821	108	7	Yes	Secreted	–	–	Motif [Y/F/W]XC; *Pst* specific
PSTG_03385	128	7	Yes	Secreted	–	–	Motif [Y/F/W]XC
PSTG_05233	126	6	Yes	Secreted	–	–	Motif [Y/F/W]XC; *Pst* specific
PSTG_06302	118	6	Yes	Secreted	–	–	Motif [Y/F/W]XC
PSTG_08956	127	6	Yes	Secreted	–	–	Motif [Y/F/W]XC
PSTG_11601	98	7	Yes	Secreted	–	–	Motif [Y/F/W]XC; *Pst* specific
PSTG_14695	152	6	Yes	Secreted	–	–	Motif [Y/F/W]XC
PSTG_19156	111	7	Yes	Secreted	–	–	Motif [Y/F/W]XC; *Pst* specific
PSTG_19591	68	4	Yes	Secreted	–	–	Motif [Y/F/W]XC; *Pst* specific

For *Yr6*, we identified 151 SNPs and 17 Indels located in 46 SP genes at the significance threshold of *P* < 1 × 10^−10^ in the phenotype and genotype correlation analysis. Most of the 46 genes had more than one SNP, and these SNPs occurred in intron, exon, or both regions (Supplementary Table 3). However, in 5 of the genes (*PSTG_00359, PSTG_09293, PSTG_09294, PSTG_14087*, and *PSTG_14482*), the variations occurred only in the promoter regions, indicating that they may have regulatory functions instead of changing the protein structures. Compared to the 46 SP genes in *Yr6*, fewer significant SPs were identified for other *Yr* genes, 2 for *Yr8*, 19 for *Yr9*, 3 for *Yr17*, and 3 for *YrTr1* (Figures 5A–E). Of the 46 SP genes in *Yr6*, 17 satisfied at least one of the five criteria used to identify effectors (Table 3). Similarly, 2 genes in *Yr8*, 5 in *Yr9* and 1 in *Yr76* were considered as avirulence candidate effectors. In addition, 21 other SP genes had significant homologies with genes in the PHI database and other 9 genes had known effector motifs.

**Figure 6:**
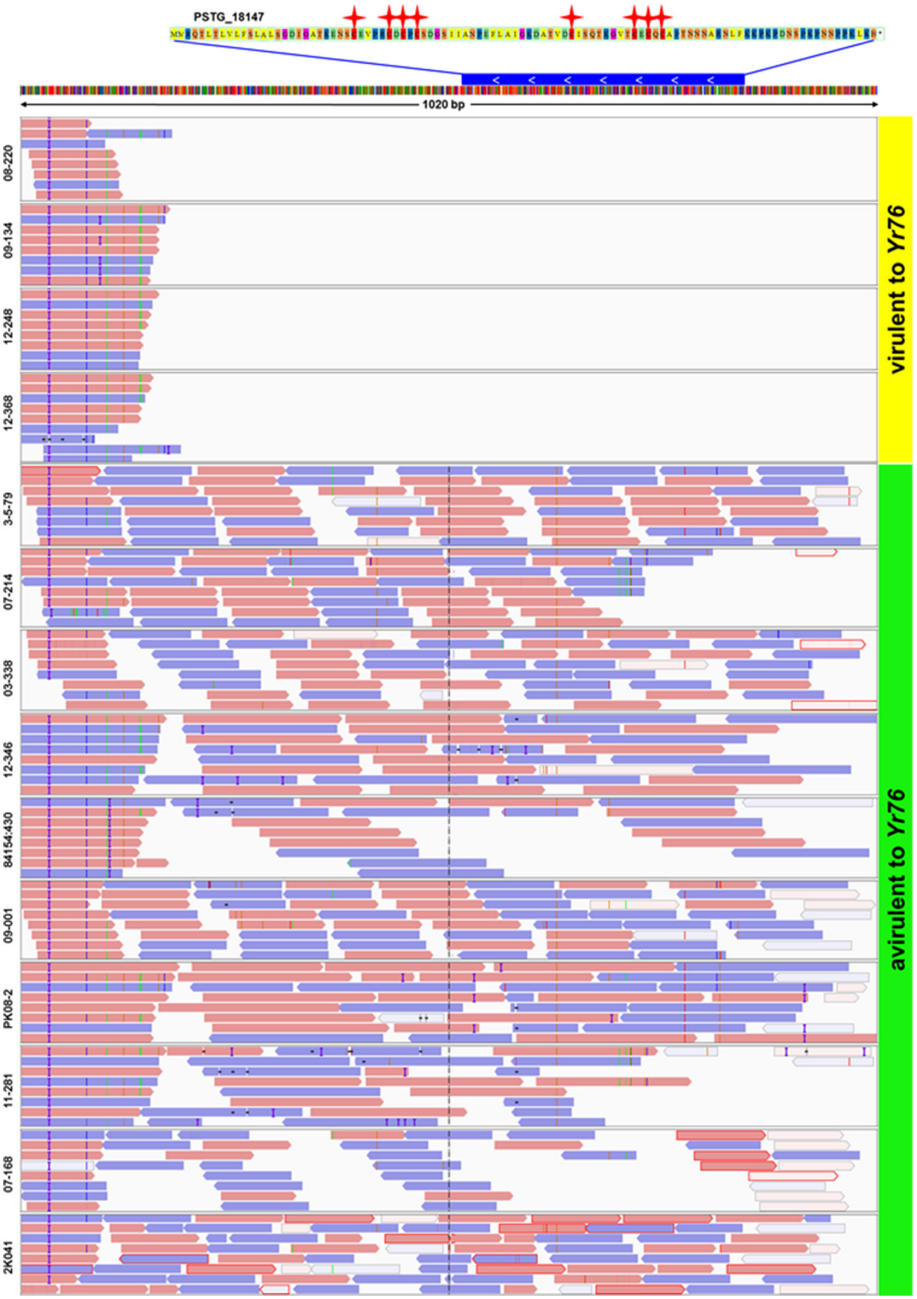
Presence of secreted protein gene *PSTG_18147* in *Yr76*-avirulent and absence in *Yr76*-virulent isolates of *Puccinia striiformis* f. sp. *tritici*. Upper: The sequence of *PSTG_18147*. Red stars represent Cysteine amino acids. Blue bar represents the gene of *PSTG_18147* and its position in Supercontig_1.1907. The white arrow shows the gene orientation. Lower: Results of mapping raw reads of 14 isolates to the PST-78 reference genome. Orange and blue bars show the reads and the orientation of mapping. Blank areas in the first four isolates indicate no reads are mapped to gene *PSTG_18147*.

In addition to the candidate effectors for the five avirulence genes, we identified a candidate, *PSTG_18147*, for *AvYr76*. Different from the SNP and/or Indel polymorphisms for in the above mentioned candidate effectors, the gene showed the presence/absence polymorphism. This *Pst*-specific gene was present in all 10 avirulent isolates but absent in all 4 virulent isolates (Figure 6). We also detected presence/absence polymorphisms for other SP genes among the isolates at the supercontig level when mapping raw reads to the PST-78 reference genome. To assess differences in genome structure, we found that some supercontigs were present in some isolates but missing in others. The number of lost supercontigs ranged from 894 for CYR32 to 2,740 for isolate 841541:430 (Supplementary Table 4). Most of presence/absence polymorphic supercontigs were relatively short (<14 Kb), and are likely unassembled chromosomal fragments rather than dispensable chromosomes often found in other fungi.

### Functional features and subcellular localizations of *Pst* SPs

**Figure 7:**
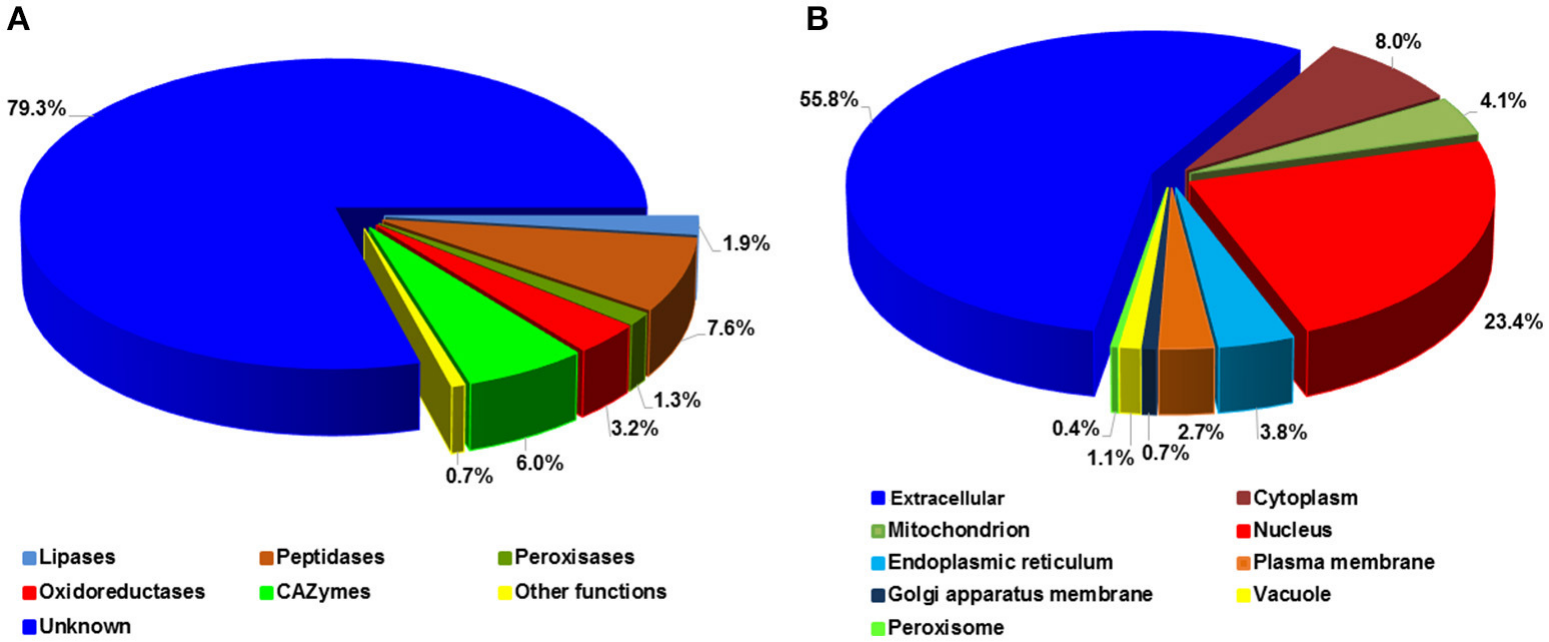
Functional annotation of *Puccinia striiformis* f. sp. *tritici* (*Pst*) secreted proteins. **(A)** Percentage distribution of the *Pst* secreted proteins with distinct enzymatic functions. **(B)** Percentage distribution of predicted subcellular locations of *Pst* secreted proteins.

To investigate the potential functions, *Pst* SPs were searched for PFAM domains (Finn et al., 2015) and further classified into five major enzyme classes, including CAZymes (Carbohydrate-Active enZymes), lipases, oxidoreductases, peptidases and peroxidases (Figure 7A). Most SPs, 1,701 out of 2,146 (79.3%), had unknown functions, as expected, with only 445 (20.7%) being annotated. Of the annotated SPs, peptidases were the largest group with 164 (7.6%) SPs, followed by CAZymes (129; 6.0%). These two enzyme classes, together with lipases (40 SPs), suggest important roles in nutrient acquisition during the *Pst* colonization in the host. We predicted the protein sub-cellular localization for each of 2,146 *Pst* SPs. The extracellular group was the largest with 1,197 (55.8) SPs. Nucleus ranked the second, harboring 502 (23.4%) SPs, and followed by the cytoplasm group (172; 8.0%) (Figure 7B). In addition, 209 proteins with nuclear localization signals were predicted to be nuclear proteins that were transported by their import machinery of the cell (Supplementary Table 3).

**Figure 8:**
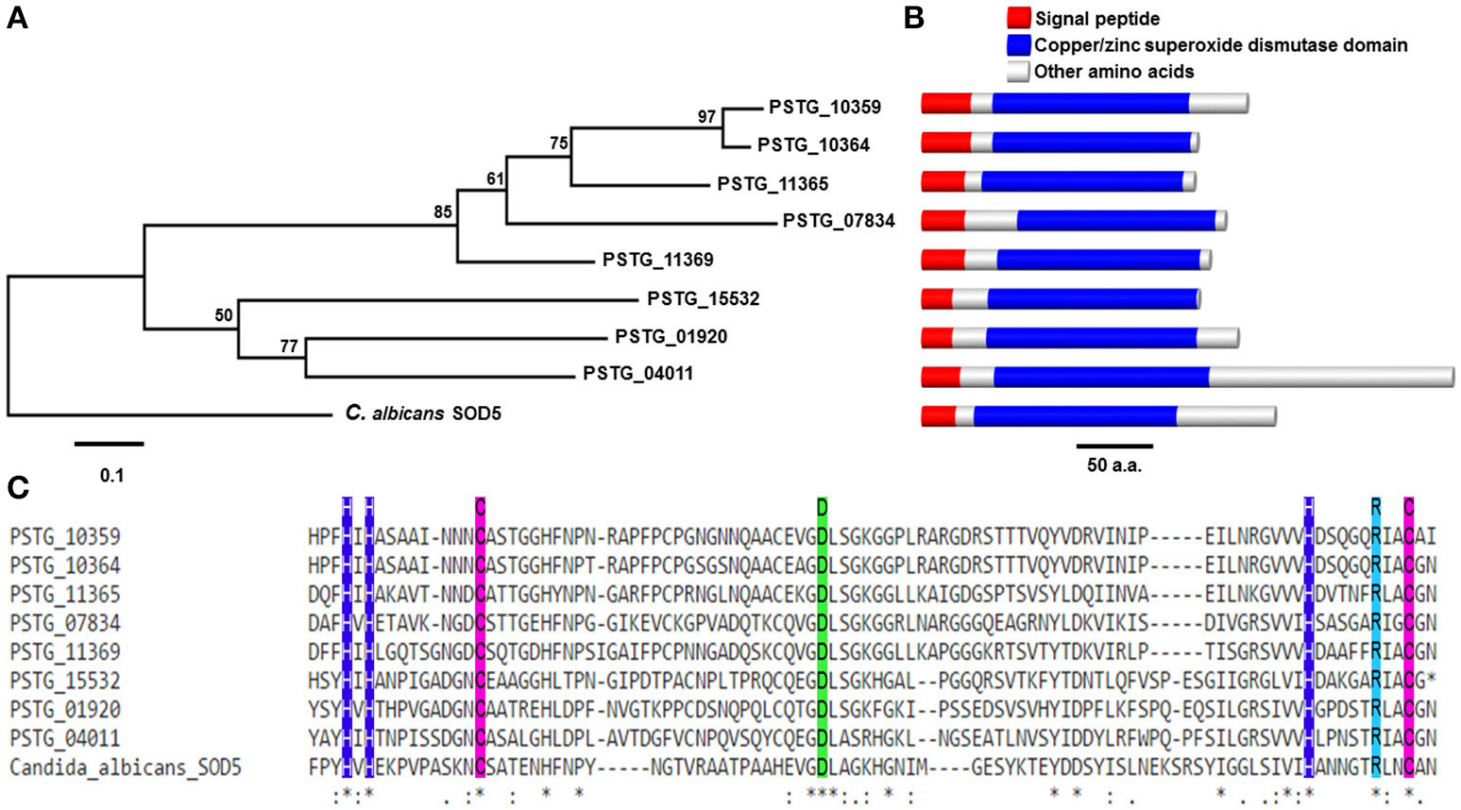
Phylogenetic and structural analysis of a class of SOD5-like secreted proteins in *Puccinia striiformis* f. sp. *tritici* (*Pst*). **(A)** Phylogenetic tree showing evolutionary relationships of eight SOD5-like secreted proteins in *Pst*. The phylogeny was estimated using the neighbor-joining algorithm, with bootstrap values indicated. The scale bar indicates an evolutionary distance of 0.1 amino acid substitution per position in the sequence. **(B)** The genomic structural of SOD5 and SOD5-like secreted proteins in *Pst*. The length of bars is scaled to the length of proteins. **(C)** Alignment of SOD5 residues F72-N164 against eight SOD5-like secreted proteins in *Pst*. Copper and zinc ligands are represented by blue and green box, respectively. The purple box represents cysteine.

Comprising of 70 (3.2%) secreted proteins, we considered oxidoreductases as an important enzyme class because their potential functions to protect the fungus against host-produced reactive oxygen species (ROS). In the further analysis, we identified 13 *Pst* SPs from the oxidoreductases to have copper/zinc superoxide dismutase (SOD) PF00080 domains, and 8 of them were homologous to the SOD5 gene in *Candida albicans* (e-value < 1e-6) (Table 3). Most of them were cysteine-rich and three were haustorial differentially expressed. The genetic relationships among the eight *Pst* SODs and the *C. albicans* SOD5 are shown in Figure 8A; their structures containing a signal peptide, a copper/zinc superoxide dismutase domain and other amino acids were illustrated in Figure 8B; and their alignment with the SOD5 residues and indication of copper and zinc ligands are given in Figure 8C.

The PHI database was searched to predict specific functions of *Pst* SPs by homology to known effector proteins (Urban et al., 2015). While 22 (10.34%) had significant hits, most SPs in *Pst* contained no known functions (Supplementary Table 3). After we applied the general effector mining criteria, a number of candidate effectors were identified, as listed in Table 3. Among these candidates, the SOD5-like proteins, as mentioned above, formed the biggest group. Another group including five SPs (PSTG_08473, PSTG_02725, PSTG_12818, PSTG_14686, and PSTG_15141) had a ring finger domain (PF13639). One (PSTG_08473) of the five SP genes was significantly correlated with avirulence to *Yr8*. Another group comprised of 3 SPs like plant avirulence determinants; PSTG_09480 and PSTG_09484 were homologous to XEG1 and PSTG_10154 was homologous to ACE1. The last group had only one protein, PSTG_02563, homologous to BcFKBP12, which is a haustorial differentially expressed secreted serine protease. In addition, we identified nine SP genes that had an effector motif ([Y/F/W]XC) and haustorially expressed (Table 3). Five (PSTG_00821, PSTG_05233, PSTG_11601, PSTG_19156, and PSTG_19591) of them were *Pst* specific. These genes were also good candidates for *Pst* effectors.

## Discussion

One of the prominent features of the present study was that we incorporated virulence phenotypes into a correlation analysis to identify avirulence effector candidates (Figure 1). A similar study was tried previously by analyzing SP gene polymorphisms between two UK isolates which were different mainly on two known *Yr* genes, and 5 candidates with single amino acid substitution were identified (Cantu et al., 2013). However, they were unable to detect other *Avr* candidates because of the incomplete virulence phenotypes and limited isolates. In another study, virulence phenotypes of 352 isolates from natural populations were used to associate SP gene polymorphisms associated with avirulence genes (Xia et al., 2016b), but the study was limited by the number of SP-SNPs used for genotyping. To overcome the limitations, we selected 14 *Pst* isolates from worldwide collections and tested them on 18 wheat lines with single *Yr* genes, which are currently used to differentiate *Pst* races (Wan and Chen, 2014; Wan et al., 2016, 2017). By doing this, we assume that there is an *Avr* gene present in the isolate that is avirulent to the single *Yr* gene. The identification of 73 *Avr* candidates corresponding to 5 *Yr* genes indicates the potential power of the correlation analysis (Figure 5).

Regarding the candidate effectors identified for the *Yr* genes, two points should be discussed. First, multiple candidates were identified as significantly correlated with an avirulence gene, even after they were filtered by the general effector mining criteria (Table 3). We suspect that some of the significant signals are false-positives and may not the causative variants since they are synonymous substitutions. The false-positives can be explained by the undetermined linkage disequilibrium (LD) due to the *Pst* clonal reproduction since the sexual recombination on alternate hosts is less important under nature conditions (Wang et al., 2015; Zhao et al., 2016). Another plausible explanation for the multiple candidates to a single *Yr* gene is that reaction to a single resistance gene may be controlled by multiple pathogen genes, as reported in powdery mildew-cereal interactions (Bourras et al., 2016) and as suggested by genetic studies of *Pst* virulence to *Yr* genes (Tian et al., 2016; Yuan et al., 2017). Second, the correlation analysis did not result in significant effectors for the rest *Yr* genes, even though they had balanced virulence-avirulence profiles, e.g., avirulence to *Yr7, Yr27, Yr43, Yr44*, and *YrExp2*. This can be caused by several reasons as discussed in Xia et al. (2016b). Here we highlighted the following four reasons. (1) The causal variations may be minor alleles which were filtered out in this study since we only kept variants having minor alleles three times or above. (2) The correlation algorithm in the present study could not take variation interactions into consideration. (3) The virulence to avirulence change may be caused by variations other than SNPs or Indels, e.g., gene presence/absence polymorphisms and copy number variations. (4) Avirulence may be controlled by non-SP genes. As our focus was on SP genes, we might miss other genes for avirulence. Therefore, future studies need to be conducted by deep sequencing more *Pst* isolates from both natural populations and artificially produced sexual populations with diverse virulence/avirulence profiles and using different algorithm models to improve the power of detecting avirulence genes.

The two nuclei in *Pst* urediniospores were suggested to be highly heterozygous in one Chinese isolate (Zheng et al., 2013) and in many US isolates (Liu et al., 2012; Cheng and Chen, 2014; Cheng et al., 2016). In the present study, we noticed different *Pst* isolates had tremendous variation in heterozygosity (Figure 3A). The highly homozygous isolates were represented by isolates 08-220 and 09-134 (race PST-127 based on the previous set of wheat cultivar differentials or PSTv-11 based on the current set of 18 *Yr* single-gene lines). The high level of homozygosity of this group was confirmed from a previous study in which 352 US isolates were genotyped with 97 SP-SNPs (Xia et al., 2016b). Isolates of this race group were distinct with other isolates based on a population study and characterized by their broadest virulence spectra and the virulence to *Yr1* and *Yr76* (Wan et al., 2016; Xia et al., 2016b; Xiang et al., 2016). This group was first detected in the western US in 2007 and became the most predominant race group in this region but not in other regions in 2009-2011. Since 2012, however, the frequency of this race group decreased due potentially to the fitness cost for maintaining the broad virulence (Chen et al., 2010; Wan and Chen, 2012, 2014; Wan et al., 2016). From this case, we speculate that *Pst* isolates with high homozygosity are less adaptive, even though they have a broad virulence spectrum. Furthermore, it seems that the heterozygosity, especially at avirulence loci, is the result of positive selection and enables *Pst* populations to evolve quickly (Hovmøller et al., 2008; Zheng et al., 2013; Cuomo et al., 2017; Yuan et al., 2017). Further studies on avirulence loci using artificially produced sexual populations and continual monitoring the natural populations are needed to fully test this hypothesis.

Our study indicated that all three wheat rust fungi have extensive of secretomes, ranging from 9.08 to 11.62% of the proteomes, which are also in the high part of the range of the plant pathogenic fungi (Lowe and Howlett, 2012). The results are consistent with the previous report for the three *Puccinia* species infecting wheat (Cuomo et al., 2017). The large secretome sizes of wheat rust fungi could be due to their complicated lifestyles: macrocyclic (the presence of all five spore stages in either natural or controlled conditions) and heteroecious (requiring of two different host species to complete the life cycle). It is presumed that different sets of SPs may be involved in the interactions between rust fungi and their cereal and alternate hosts (Krijger et al., 2014). Other rust fungi in Pucciniales also showed such relationship. For example, the heteroecious poplar leaf rust fungus *Mlp* has 11.2% SPs (1,829 secreted over 16,399 proteins) (Duplessis et al., 2011), whereas only 4.9% (802 of 16,271 proteins) were predicted as SPs for autoecious flax rust fungus *Mli* (Nemri et al., 2014).

Moreover, the SSSPs are enriched in wheat rust secretomes. This is similar to many biotrophs and symbionts that intimately interact with their host cells. The functions of most SSSPs (90%) are unknown since they do not have defined domains. However, a kingdom-wide analysis of fungal secretomes suggested that the number of SSSPs could correlate with their proteome size, lifestyle and taxonomic position (Kim et al., 2016). Generally, biotrophs, e.g., Pucciniomycotina and Ustilagomycotina species, have the highest number of SSSPs. Given the similar lifestyles and taxonomic positons of the three wheat rust fungi, we speculate that these SSSPs might be impacted by different evolutionary histories compared with conserved ones, e.g., during the coevolutionary arms-race between rust fungi and wheat hosts (Krijger et al., 2014; Kim et al., 2016). Therefore, these SSSPs coding genes may provide new insight in understanding how rust fungi interact with their primary wheat host, auxiliary grass hosts and alternate hosts (Zhao et al., 2016).

Since the draft genome of *Pst* was available in 2011, from our knowledge, only few genes (or their products) have been validated as fungal virulence effectors, and functionally involved in host-pathogen interactions (Cheng et al., 2015; Liu et al., 2016; Ramachandran et al., 2017). All of these validated effectors suppress host-produced ROS. In the present study, we identified 70 secreted proteins as oxidoreductases, an important enzyme class with potential functions to protect the fungus against host-produced ROS (Chi et al., 2009). By searching the PHI database, we identified 13 SPs in *Pst* highly homologous to the SOD5 gene in *Candida albicans*, which was reported to be associated with pathogenicity of this human pathogen (Martchenko et al., 2004). In *C. albicans*, SOD5 is an extracellular protein with an open copper site, and could accumulate outside the cell in an inactive form that subsequently utilizes host copper to transform itself into an antioxidant defense for the pathogen (Gleason et al., 2014). Therefore, these SOD5-like SPs in *Pst* could be a common class of virulence effectors for its defense against plant basal immunity, since suppression of ROS accumulation has been observed by cloned virulence effectors for successful *Pst* infection (Liu et al., 2016). Several SP genes in *Pst* may be related to pathogenicity or trigger hypersensitive response as they are homologous to XEG1, ACE1, or CaRING1. These proteins have been reported to be plant avirulence determinants in *Phytophthora sojae* (Ma et al., 2015) and *Magnaporthe oryzae* (Böhnert et al., 2004), or required for cell death and the salicylic acid-dependent defense response in *X. campestris* pv. *vesicatoria* (Lee et al., 2011). Nine SP genes of *Pst* identified in the present study had an effector motif ([Y/F/W]XC). This conserved motif is present in many fungal pathogens including *Blumaria graminis* (Godfrey et al., 2010) and *Melampsora larici-populina* (Duplessis et al., 2011). The PSTG_02563 protein identified in the present study was highly homologous to BcFKBP12, which is involved in the sulfur repression of the synthesis of a secreted serine protease in *Botrytis cinerea* (Meléndez et al., 2009). Particularly, this gene was haustorial differentially expressed. Some candidates for specific *Avr* genes in *Pst* are likely related to pathogenicity or virulence as they have homology to genes in other fungi with similar functions. For example, *PSTG_09464*, which was associated with *AvYr6*, is homogous to *ZtCBR1*, a virulence gene in *Zymoseptoria tritici* (Derbyshire et al., 2015). *PSTG_16061*, also associated to *AvYr6*, has a CFEM domain that has been reported for fungal pathogenesis (Kulkarni et al., 2003). *PSTG_16854*, a candidate SP gene for *AvYr9*, has a fuz7 protein kinase domain, which has been reported as a virulence factor in *Ustilago maydis* (Lanver et al., 2010). Therefore, these genes are excellent candidate effectors with priority for further functional validation.

The mature methods for testing the function of effector candidates include (a) delivery of effector candidates to plant cell through bacteria type III secretion system to test the ability to induce hypersensitive response or to suppress pattern triggered immunity (Yin and Hulbert, 2011), and (b) use of host-induced gene silencing to test the involvement of candidates in fungal pathogenesis (Yin et al., 2015). We speculate that these methods can also be used to test the avirulence function of some *Pst* candidate effectors identified in this study. Once the candidate *Avr* genes are confirmed and their functions are determined, the information will shed the light on the *Pst*-wheat interactions, and the *Avr* genes can be used to develop gene-specific markers for monitoring the pathogen population changes and identify effective resistance genes from germplasm of wheat and related species using an effectoromics approach as suggested by Vleeshouwers and Oliver (2014).

## Author contributions

CX, MW, DJ, and DS participated in isolates collection, genomic DNA extraction, and Ion Proton sequencing. CX, OC, and XC analyzed and interpreted the data. CX was a major contributor in writing the manuscript. XC conceived the study, designed the experiments and wrote the manuscript. All authors reviewed and approved the final manuscript.

### Conflict of interest statement

The authors declare that the research was conducted in the absence of any commercial or financial relationships that could be construed as a potential conflict of interest.
